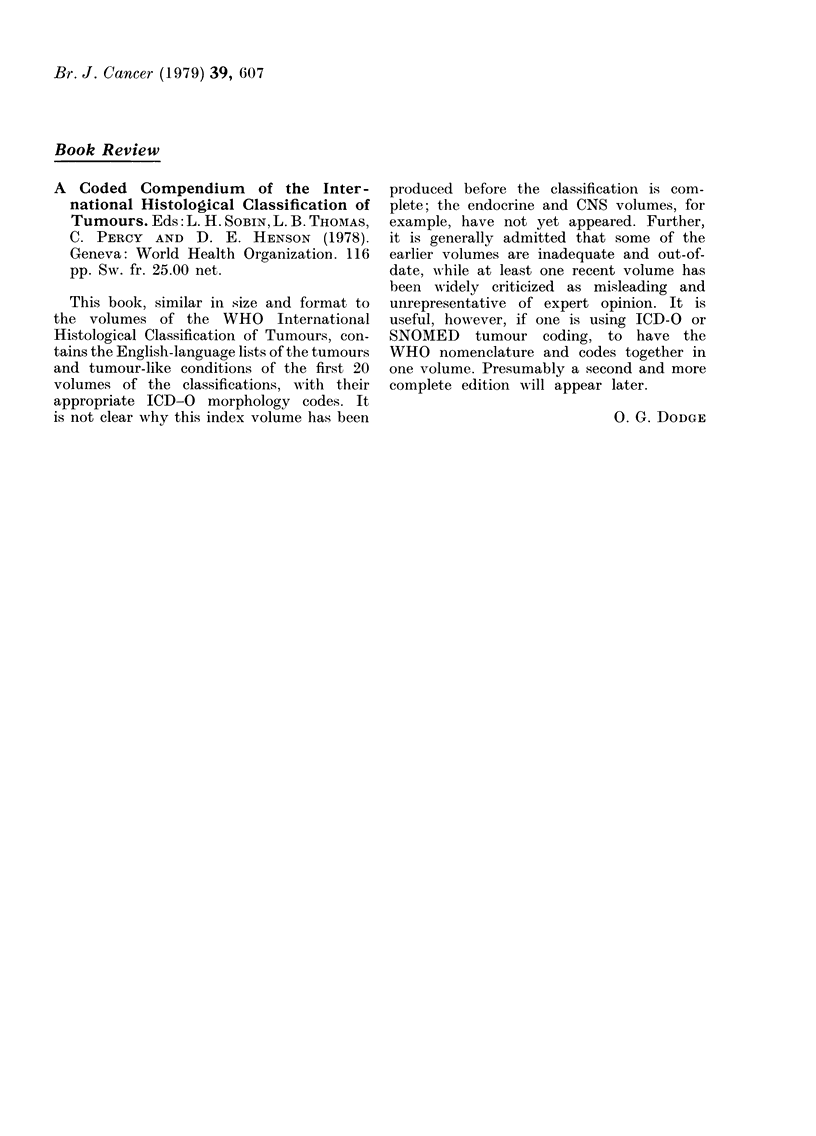# A Coded Compendium of the International Histological Classification of Tumours

**Published:** 1979-05

**Authors:** O. G. Dodge


*Br. J. Cancer* (1979) **39**, 607

### *Book Review*

**A Coded Compendium of the International Histological Classification of Tumours.** Eds: L. H. Sobin, L. B. Thomas, C. Percy and D. E. Henson (1978). Geneva: World Health Organization. 116 pp. Sw. fr. 25.00 net.

This book, similar in size and format to the volumes of the WHO International Histological Classification of Tumours, contains the English-language lists of the tumours and tumour-like conditions of the first 20 volumes of the classifications, with their appropriate ICD–O morphology codes. It is not clear why this index volume has been produced before the classification is complete; the endocrine and CNS volumes, for example, have not yet appeared. Further, it is generally admitted that some of the earlier volumes are inadequate and out-of-date, while at least one recent volume has been widely criticized as misleading and unrepresentative of expert opinion. It is useful, however, if one is using ICD-O or SNOMED tumour coding, to have the WHO nomenclature and codes together in one volume. Presumably a second and more complete edition will appear later.

O. G. Dodge